# News and Notes

**Published:** 1912-06

**Authors:** 


### NEWS AND NOTES

### PRELIMINARY PROGRAM FOR THE TWELFTH SEMI-ANNUAL SESSION OF THE CHATTAHOOCHEE VALLEY MEDICAL AND SURGICAL ASSOCIATION, TO BE HELD AT ATLANTA, GA., JULY 16TH AND 17TH, 1912.

Call to Order—11 A. M.

Invocation—By Rev. A. R. Holderby, Atlanta, Ga.

Address of Welcome on Behalf of the City of Atlanta—By Hon. Thomas H. Jeffries, Atlanta, Ga.

Address of Welcome in Behalf of Fulton County Medical Society—By Dr. J. Scott Todd, Atlanta, Ga.

Response in Behalf of the Chattahoochee Valley Medical and Surgical Association—By J. N. Baker, M. D., Montgomery, Ala.

Personal Observations upon the Effects of Treatment of Chronic Constipation—L. M. Gaines, M. D., Atlanta, Ga. Leaders in discussion: G. S. Murray, G. M. Saliba, C. L. Williams.

The relation of the Nervous System to Tuberculosis—J. Cheston King, M. D., Atlanta, Ga. Leaders in discussion: B. L. Wyman, E. C. Thrash, Hansell Crenshaw.

The Operative Treatment of Fractures in and about Joints—By H. P. Cole, M. D., Mobile, Ala. Leaders in discussion: Jno. D. S. Davis, Michael Hoke, William P. Nicolson.

AFTERNOON SESSION, 2 P. M.

Two Unusual Cases of Choroiditis—By Richard R. Daly, M. D., Atlanta, Ga. Leaders in discussion: T. E. Mitchell, Geo. H. Cooper, W. G. Harrison.

Some Observations on the Use of Apormorphia—By J. S. Horsley, Sr., M. D., West Point. Leaders in discussion: S. A. Visanka, J. S. Todd, C. L. Williams.

Arthitis Deformans—N. B. Dean, M. D., Alexander City, Ala. Leaders in discussion: Theo. Toepel, L. C. Morris, H. S. Bruce.

The Importance of a Complete Eye Examination for Diagnostic Purposes—By Hugh M. Lokey, M. D., Atlanta, Ga. Leaders in discussion: B. F. Rea, H. S. Persons, Geo. M. Chambers.

Major Surgery in Rural Homes—H. T. Hamner, M. D., Camp Hill, Ala. Leaders in discussion: J. A. Goggans, Thos. F. Taylor, F. M. Ridley, Sr.

The Value of the X-Ray in Diagnosis—By Arch B. Elkin, M. D., Atlanta, Ga. Leaders in discussion: H. R. Slack, Velpeau Langley, H. P. Cole.

The Surgical Aspects of Visceroptosis—By J. N. Baker, M. D., Montgomery, Ala. Leaders in discussion: Gaston Torrance, J. C. Johnson, J. L. Bowman.

Salvarsan, Neosalvarsan—914: 745 Administrations—By E. G. Ballenger, M. D., Atlanta, Ga. Leaders in discussion: H. P. Cole, Jno. D. S. Davis, W. F. Shallenberger.

Malaria Clinically Considered—By H. G. Moore, M. D., Opelika, Ala. Leaders in discussion: W. T. Langley, J. J. Winn, J. H. McDuffie.

Injuries to the Spinal Cord—F. K. Boland, M. D., Atlanta, Ga. Leaders in discussion: B. L. Wyman, J. L. Bowman, W. D. Gaines.

Some Observations on the Use of Veratrum Viride—By J. P. Motley, M. D., Wadley, Ala. Leaders in discussion: Thos. Northern, J. S. Todd, J. S. Horsley, J. M. Welch.

The State's Need of Medical Inspection of Schools—By H. C. Miller, M. D., Atlanta, Ga. Leaders in discussion: H. W. Terrell, W. H. Sanders.

Arterio Schlerosis—By R. F. Elrod, M. D., Cottonton, Ala. Leaders in discussion: Cyrus M. Strickler, J. L. Bowman, T. H. Street.

After-Treatment of Surgical Cases—By J. L. Campbell, M. D., Atlanta, Ga. Leaders in discussion: H. T. Hamner, H. S. Munroe, H. B. Disharoon.

Importance of Medical Organization—By A. L. Harlan, M. D., Alexander City, Ala. Leaders in discussion: L. W. Johnson, E. C. Davis, H. F. Harris.

MORNING SESSION—SECOND DAY.

Call to Order—8 A. M.

Entero Collitis in Children—By Velpeau Langley, M. D., Opelika, Ala. Leaders in discussion: C. E. Boynton, R. T. Dorsey, J. M. Welch.

Opthalmia Neonatorum the Most Prolific Cause of Preventable Blindness—By H. S. Persons, M. D., Montgomery, Ala. Leaders in discussion: R. R. Daly, Geo. H. Cooper, Hugh M. Lokey.

The Status of Deaf Mutism—By M. M. Stapler, M. D., Macon, Ga. Leaders in discussion: G. Dunbar Roy, W. G. Harrison, Frank Thigpen.

Pin Point Os Uteri—John Prather, M. D., Fort Davis, Ala. Leaders in discussion: W. F. Shallenberger, J. R. B. Branch, R. S. Hill.

Early Diagnosis of Diseases of the Gall Bladder—By Gaston

Torrence, M. D., Birmingham, Ala. Leaders in discussion: Willis Jones, E. G. Jones, J. L. Campbell.

The Use of the Laboratory in the Diagnosis and Treatment of Disease—By C. W. Gould, M. D., Atlanta, Ga. Leaders in discussion: E. C. Thrash, Jno. S. McLester, G. S. Murray.

Personal Observations in Treatment of Typhoid Fever—By Onslow Reagan, M. D., Alexander City, Ala. Leaders in discussion: Thos. Northen, J. J. Winn, E. P. Green, P. M. Poer.

Some Lessons Learned in Obstetrical Experience—By J. H. McDuffie, M. D., Columbus, Ga. Leaders in discussion: J. S. Horsley, W. W. Stevenson, J. M. Welch.

A Consideration of Post Operative Peritoneal Adhesions—By J. A. Goggans, M. D., Alexander City, Ala. Leaders in discussion: E. G. Jones, H. P. Cole, W. F. Westmoreland.

Fractured Patella, Report of Case—W. L. Cooke, M. D., Columbus, Ga. Leaders in discussion: Willis Westmoreland, Hugh McCullough, F. M. Ridley, Jr.

Diabetis Mellitus—By C. S. Yarbrough, M. D., Auburn, Ala. Leaders in discussion: C. W. Gould, W. W. Stevenson, J. S. Horseley.

The Management of Typhoid Fever in the Country—By C. O. Griffin, M. D., Alexander City, Ala. Leaders in discussion: G. H. Moore, T. H. Haralson, John Prather.

AFTERNOON SESSION—SECOND DAY.

Call to Order, 2 P. M.

Surgical Aspects of Pellagra—By Dr. ____________ Glenn, Columbus, Ga. Leaders in discussion: Stewart Roberts, G. C. Mizell, H. P. Cole.

Intestinal Obstruction—By H. S. Monroe, M. D., Columbus, Ga. Leaders in discussion: E. G. Jones, L. L. Hill, Willis F. Westmoreland.

The Eye as an Index to Disease—By Park Howell, M. D., Atlanta, Ga. Leaders in discussion: Hugh M. Lokey, H. S. Persons, W. G. Harrison.

Diagnosis of Gastro-Intestinal Diseases by Means of the X-Ray—By C. R. Andrews, M. D., Atlanta, Ga. Leaders in discussion: Gaston Torrance, J. N. Baker, Velpeau Langley.

Some Observations on Treatment of Acute Intestinal Diseases of Infants—By Thos. Northen, M. D., Ashland, Ala. Leaders in discussion: J. M. Poer, L. M. Gaines, J. H. McDuffie.

A Case of Erythrometalgia—By G. Stewart Murray, M. D., Columbus, Ga. Leaders in discussion: Hansell Crenshaw, E. D. Bondurant.

Papilloma of Bladder—By Montague L. Boyd, M. D., Atlanta, Ga. Leaders in discussion: R. S. Hill, J. R. B. Branch, W. L. Cooke.

Endometritis—By W. F. Shallenberger, M. D, Atlanta, Ga. Leaders in discussion: J. R. B. Branch, Hugh McCullough, J. H. McDuffie.

LAST SESSION.

The Neglected Prostate—By W. L. Champion, M. D., Atlanta. Leaders in discussion: W. A. Person, E. P. Merritt, W. B. Emory.

Entero Collitis in Children—By J. D. Thompson, M. D., Toomsboro, Ga. Leaders in discussion: Thos. Northen, C. A. Rhodes, C. F. Benson.

---

### CRAWFORD W. LONG.

BY JOHN CHALMERS DA COSTA, M. D., LL. D., GROSS PROFESSOR OF SURGERY IN JEFFERSON MEDICAL COLLEGE.*

*(Continued from Last Issue.)*

Now and then a real leader, an original force, a truly great man comes into the world, and moves us as one inspired. He dares to lift the veil which hangs before the mysteries, the veil which lesser men are too ignorant to observe, too indifferent to regard, or too cowardly or incapable to displace. Such a man seeks truth and scorns wealth—courts labor and forgets ease—fights dragons and slays giants—is the slave to duty, is contemptuous of popularity, and finally wrings

"the secret of deliverance forth
Whether it lurk in hells or hide in heavens."

He originates. Every institution, says Emerson, "was once the act of a single man."

All such men have earned the reverent love and the eternal gratitude of humanity. Love and gratitude are the debts men owe to the memories of the heroes of progress, because of their labors, pains, perils and sacrifices. What would have become of the world without such men?

"Men
Perished in winter-winds till one smote fire
From flint-stones coldly hiding what they held,
The red spark treasured from the kindling sun.
They gorged on flesh like wolves, till one sowed corn,
Which grew a weed, yet makes the life of man:
They mowed and babbled till some tongue struck speech,
And patient fingers framed the lettered sound.
What good gift have my brothers, but it came
From search and strife and loving sacrifice?"

The world is often ignorant of its greatest men. Men, to us nameless, made some of the grandest discoveries and perfected some of the most remarkable inventions.

"Who found the seeds of fire and made them shoot,
Fed by his breath, in buds and flowers of flame?
Who forged in roaring flames the ponderous stone,
And shaped the moulded metal to his need?
What gave the dragging car its rolling wheel,
And tamed the steed that whirls it circling round?
All these have left their work and not their names."

When a man has found a radiant truth, has done some gleaming deed, but has received no tribute of praise or glory, it is a peculiarly grateful thing to see the conscience of the world awaken, and to find men place the name of their long neglected benefactor

"On Fame's eternal beadroll."

Seventy years ago to-day, on the 30th of March, 1842, and in the little village of Jefferson, Jackson county, Georgia, anaesthesia was first intentionally produced to permit of the painless performance of a surgical operation. This discovery was one of the greatest in the history of science and ranks in importance with the discovery by Harvey of the circulation of the blood—

by Franklin of phenomena of electricity, by Jenner of vaccination, by Pasteur of bacteriology, and by Lister of antiseptic surgery. The giving of ether as a surgical anaesthetic was not a haphazard accident, but was reasoned out from observations.

The man who first gave ether for surgical purposes was Crawford W. Long, a native and resident of the State of Georgia, and a graduate of the University of Pennsylvania in the class of 1839. There seems a peculiar adjustment to the eternal fitness of things in the fact that a son of the University founded by the great philosopher, Benjamin Franklin, should have made one of the greatest practical discoveries of all time.

Long's discovery was not made in a splendidly equipped institution of world-wide fame, nor by a professor whose lecture room was packed with eager students, but by a modest, unassuming country doctor, dwelling in an isolated village. Truly! greater things for mankind have come from the hut than from the palace, from the peaceful country than from the roaring town.

We meet to-day in commemoration and celebration: in commemoration of the discovery of ether anaesthesia, and in celebration of the noble achievement of a great son of this grand old school.

We will strive to:

> Part the mists which almost hide
> A man of former days
> And spin upon the Wheel of Truth
> Some golden threads of praise.

No one disputes that Long gave ether for surgical purposes over four years before Morton did, and at least two years before Horace Wells pulled the tooth of a patient who was under the influence of Nitrous Oxide Gas. There is no claim that Morton knew anything of Long's observations. It is freely admitted by all that Warren, in the operating room of the Massachusetts General Hospital, gave to Morton the opportunity to dramatically impress the world with his views. Morton and Warren made the world hear. Long made the discovery, and would also have made the world hear had he had a great City Hospital as a forum from which to speak, and a celebrated surgeon as a spokesman and advocate. Long has been criticized for not publishing his discovery at once. Jenner waited twenty years to publish his and after twenty years had only made twenty-three observations. Suppose someone had published about vaccination after Jenner had worked nineteen years, would Jenner any the less have been the discoverer?

Long made no official claim to the discovery until 1849, when he told his story to the Medical Society of Georgia. He did so then only because his friends thought he would be doing himself injustice to keep silent. His intention had been to collect enough cases to thoroughly test the method. This was slow work in a country district in which surgical operations were few and far between. He used ether seven or eight times in four years. In December, 1846, he read of Morton's success. Soon after Morton, Jackson and Wells became involved in a bitter controversy and Long shrunk from such things and abhorred the patenting of ether.

In the statement to the Georgia Medical Society, Long presented an affidavit of James M. Venable, then living, stating that ether had been given to him by Long on two occasions in the Spring of 1842—an affidavit of Andrew J. Thrumond stating that he saw Long do one of the operations on Venable—affidavits of E. S. Rawls and Wm. H. Thrumond declaring that they witnessed one or both operations, and other conclusive evidence. The original affidavits still exist.

Morton patented ether in 1846 under the name of "letheon." Wells opposed Morton's patent, went insane, and committed suicide in 1848. The government never enforced the patent right and army surgeons used ether freely in the Mexican war, Morton getting no return for it. In 1849 Morton applied to Congress for a grant of $100,000 as compensation for his losses and reward for his alleged discovery. Jackson opposed Morton's claim. Jackson claimed that he had suggested ether to Morton.

The controversy was acrimonious and protracted. In 1852 the French Academy of Sciences granted a prize to Jackson as the discoverer of ether, and a like amount to Morton, as the first to apply it.

In 1854 Dr. Long was persuaded to write a letter to Senator Dawson of Georgia telling the story of the discovery in 1842. Jackson had a conference with Long and finally withdrew his own claim in Long's favor. In the "Boston Medical and Surgical Journal," April 11, 1861, will be found a letter from Jackson giving Long the credit. No money was ever granted by the Government.

Jackson, like Wells, went insane, and died in 1880. Morton died in 1868, getting an apoplexy while enraged at learning of attempts to deprive him of the glory of the discovery. Long, free from such heart-burnings, pursued the calm ways of a country doctor, and made no further attempt to establish his claim. He led a useful and happy life and died in 1878. Morton, probably because men thought that the Government had treated him shabbily, came to be regarded as the real discoverer, and until 1877 there was no one to say nay. In that year, Dr. J. Marion Simms published an article in the May number of the "Virginia Medical Monthly" claiming that Long had made the discovery. There is one serious mistake in the article of Dr. Simms. He stated that S. C. Wilhite, a student of Long, suggested to his preceptor the use of ether.

Wilhite was not with Long in 1842, in fact, did not go with him until 1844. Wilhite corrected this error in a letter to Dr. Long, dated June 27, 1877. Professor Gross in commenting on Simms' paper (System of Surgery), says: "Although he (Long) may have been, and probably was, the first to use this drug as a means of preventing pain, he failed to interest the profession in it and has thus lost all just claim to the honor of one of the greatest discoveries ever achieved by human genius." Professor Agnew must have felt as Gross did, for in his book (Principles and Practice of Surgery), he gives Morton the credit for the discovery and does not even mention the name of Crawford Long. The claims of Long have since found able champions in Sir James Paget, Dr. George Foy, of Dublin; Dr. Hugh H. Young, of Baltimore; Dr. Isham H. Goss, of Athens, Georgia; Dr. Luther Grandy, and Rose Pendleton Chiles. Dr. Frances R. Packard tells the story very impartially in his admirable "History of Medicine in the United States," which was published in 1901.

Frederck W. Hewitt in his work on "Anaesthetics" (1901) says: "There seems to be no reasonable doubt that in 1842 Dr. Crawford W. Long, a country practitioner of Jefferson, Jackson County, Georgia, United States of America, administered ether vapor with the distinct object and fortunate result of producing insensibility to pain during a surgical operation whch he performed, and that he subsequently employed the same means with equal success." Henry M. Lyman in Ashhurst's International Encyclopaedia of Surgery (1889) says that Long gave ether in 1842, but as he "resided in a remote and isolated portion of the country, and as he published no statement of his experience, his discovery remained unknown."

Of late years Long's claims have been more and more regarded until justice at length prevails.

The Medical Society of Georgia has erected a monument to Long in Jefferson, where ether was first used as an anaesthetic. The Legislature of Georgia has resolved to place his statue, with that of Alexander H. Stephens, in the statuary hall of the National capitol.

On December 1, 1911, Dr. Dudley W. Buxton, the distinguished English anaesthetist, presented to the Section of Anaesthetics of the Royal Society of Medicine an article which seems final and conclusive. It is written with that literary grace and painstaking accuracy which characterize all of Dr. Buxton's productions. In this article will be found a resumé of Long's life—the story of the discovery and reproductions of various convincing documents; among them are: A letter from Dr. Long to R. H. Goodman (dated February 1, 1842), ordering the ether for the first operation, and a covering letter from Goodman—affidavits of James M. Venable and others, previously referred to—Long's bill to Venable charging twenty-five cents for the ether used and two dollars for the operation—extract from Long's record book of the operation done on Venable and charge for the operation and ether used. It is dated March 30, 1842. Certificate of Mary Vincent and her husband, declaring that Long gave Mrs. Vincent ether in 1843. There are also copies of other important and interesting papers. Buxton's complete and masterly study may be read in the published Proceedings of the Royal Society of Medicine, January, 1912. It gains greater emphasis by coming to us across the sea from a gentleman free of any possible prejudice or partiality. It is the unvarnished truth, and the world now regards Long as the real discoverer. Hence I do not stand here courting controversy. I am not obliged to search dusty records in order to clear up controverted points. I do not need to delve deep in obscure mines after the nugget of Truth. Simms, Young, Buxton and others have found that nugget and the gleaming metal may be seen and can be tested by all men.

Crawford Williamson long was born in Danielsville, Madison County, Georgia, November 1, 1815. His family was prominent socially and in public affairs.

Crawford's grandfather was Captain Samuel Long, an Irishman by birth and an adopted son of Pennsylvania, who resided in Carlisle. He married Miss Williamson of Ulster, Ireland; served in the army of Washington and at the Yorktown surrender, was a captain in the command of Marquis de Lafayette. At the termination of the war with the Mother Country, he became a citizen of Georgia. His son James Long was a planter and was for four years Clerk of the Supreme Court. He sat in the State Senate for two terms, and was the intimate and trusted friend of the celebrated statesman, Wm. H. Crawford, a man who was successively United States Senator, President pro tem. of the Senate, Minister to France, Secretary of the Treasury, and candidate for Presidency of the United States in 1824, against John Quincy Adams, Andrew Jackson and Henry Clay. The subject of this address was named Crawford, after the great statesman and Williamson after Captain Long's wife. Dr. Long's mother, Eliza Ware, was a Virginian, and an energetic warm-hearted, ambitious, sympathetic woman, of refined taste and much literary ability.

As a boy Crawford was educated in the Academy of his native town. He was bright, interesting, studious and lovable. He was an entirely normal boy and loved dogs, horses, fishing, shooting, and out-door sports. He entered Franklin College (now the Department of Liberal Arts of the University of Georgia) and graduated when only nineteen years of age, taking the second honor. At college he formed a friendship, which was to last a life-time, with Alexander H. Stephens, a man destined to become Vice-President of the Southern Confederacy.

After graduating, he studied for a time under a preceptor, and then took a course of medical lectures in Transylvania University. This school was in Lexington, Kentucky. Long rode on horseback from Georgia to Kentucky, crossing rugged mountains and passing through regions not yet free from treacherous Indians. In the fall of 1837, he went to Philadelphia and entered as a medical student in the University of Pennsylvania, from which institution he graduated in 1839. Agnew graduated in 1838. The two boys must have known each other and have often ridden out together from the University to Blockley Hospital While Long was in Philadelphia he resided in a Quaker household at the corner of 19th and Market streets. When Long went up to college seventy-five years ago the United States was a small country compared with the mighty nation which now reaches into the very portals of the distant sunset. There were twenty-six states and two territories (Florida and Wisconsin). Most of the vast region beyond the Mississippi, out of which twenty imperial commonwealths have been made, was a wilderness haunted by wild Indians and infested by savage beasts. Much of it belonged to Mexico. Texas was a republic and Samuel Houston was its president. The population of the country numbered about fifteen million people and approximately one-fifth of them were slaves. Martin Van Buren was President of the United States, and Richard M. Johnson was Vice-President. The navy list still held the names of those old heroes, Rogers, Barron, Stewart and Hull. Winfield Scott was a brigadier-general in charge of the department of the East.

Roger B. Taney was Chief Justice of the Supreme Court and Joseph Story sat by his side. There was no national debt and the Government was preparing to distribute a surplus of thirty-seven millions dollars among the States. There were 1,600 miles of railroad in operation in the country and 120 miles in Pennsylvania.

In the United States Senate sat Franklin Pierce, Daniel Webster, Silas Wright, James Buchanan, Thomas Clayton, William C. Rives, John C. Calhoun, Wm. R. King, Robert J. Walker, John J. Crittenden, Henry Clay and Thomas H. Benton.

In the House of Representatives were John Quincy Adams, Caleb Cushing, Millard Filmore, John Sergant, Henry A. Wise, John Bell, James K. Polk and Thomas Corwin.

To send a one-sheet letter for over 400 miles cost twenty-five cents—from Philadephia to New York ten cents—not over thirty miles six cents.

Truly it is a far cry from the United States of the time of Van Buren to the United States of the time of Taft.

The University of Pennsylvania was first in renown among the twenty-eight medical schools of the country, and possessed the ablest faculty in the United States. The buildings were at 9th and Chestnut streets, where the Post Office now stands. On the rolls of the University were 400 medical students over one-seventh of the entire number in the land.

Philip Syng Physick, the pupil of John Hunter and the father of American surgery, died during Long's first course. At the time of his death he was Emeritus Professor of Surgery and Anatomy. He was the first to use catgut as a ligature material—devised the stomach tube and many useful instruments—and advised the treatment of ununited fracture by the seton. A specimen of a fractured humerus successfully treated by Physick is to be seen to-day in the museum. For months after his death and by his direction his grave was guarded to keep away resurrection men, as he had a great horror of being dissected.

William Gibson, the pupil of Sir Charles Bell, was the Professor of Surgery. He had served under Wellington in Belgium and was wounded at Waterloo. In 1819 he was called from the University of Maryland to succeed Physick in Pennsylvania. He was the first man to tie the common iliac artery (1812). He twice did a successful Caesareon Section on the same patient, and saved the mother and both children. Nathaniel Chapman, the wit, critic, booklover, social light, jovial companion, and scientist, was Professor of Physic and Clinical Medicine. He stood without a peer as a practitioner and in spite of a congenital speech defect, was one of the greatest teachers in America.

Chapman's book on Therapeutics was widely celebrated.

George B. Wood, the profound scholar, the keen observer, the original thinker, taught Materia Medica. With Franklin Bache he edited the United States Dispensatory. For many years he practically determined the views of the whole profession on ethics and practice. His lectures were the pride and glory of the University and had immense influence in moulding the minds of the students. No man who has held a chair in the University brought it greater reputation than did George B. Wood. His condemnation of the premature reporting of cases and drug actions may well have decided Long a few years later to delay in publishing a report of the actions of ether. Wood spoke of immature views and premature judgments as ignes fatui. He insisted that observers must never be content with a single experiment. (See Introductory lecture 1840.)

William E. Horner, he of the feeble frame, melancholy temperament, scholarly faculty and original bent, was Professor of Anatomy. He is particularly remembered as the founder of St. Joseph's Hospital and the discoverer of the tensor tarsi, which is still called Horner's muscle. Samuel Jackson, who did so much to introduce the principles of Laennec and Louis to the American profession, was Professor of Institutes of Medicine.

Hugh L. Hodge, who had been forced to abandon a surgical career because of impaired sight, was Professor of Midwifery, having defeated Charles D. Meigs for the chair. Hodge's forceps and pessaries were known all over the world.

Robert Hare was the celebrated Professor of Chemistry. He had been a fellow-student of Silliman, and when only twenty years of age, had invented the oxyhydrogen blowpipe. He was called to the University from William and Mary College. He was one of the ablest chemists and electricians then living, was a most impressive lecturer and a highly successful experimenter.

Such were the men of the Faculty of '38 and '39, the men to whom the young Georgia student listened, the men who helped to guide and direct his mind. The session began November 1st, according to the catalogue; it ended "about the first day of March ensuing." Commencement was evidently a movable feast, for the catalogue states that it is "held generally about the first of April." No text books were recommended in the catalogue, but we know that students used the "Syllabus of Wood's Lectures," "Chapman's Therapeutics," "Gibson's Surgery," "Chapman's Therapeutics," "Gibson's Surgery," "Horner's Anatomy" and "Hare's Chemistry."

Blockley stood where it does now, and some of the buildings are very little changed externally. Agnew says that at this period, Blockley was "the greatest clinical school of the country." Every Saturday morning many busses gathered at Ninth and Chestnut, and crowds of students rode out to clinical lessons within those grim walls. Lectures were given by Samuel Jackson, Robley Dunglison, Joseph Pancoast and William Gerhard. J. M. DaCosta speaks of Gerhard as "the greatest observer and clinician America has produced."

In those days, William Norris, George B. Wood, John Rhea Barton and John K. Mitchell were at the Pennsylvania Hospital.

Samuel D. Gross had not yet gone to Louisville from Cincinnati, and George McClellan was serving his last year as Professor of Surgery in Jefferson. Students of those days were far more turbulent than now, and fierce combats were common between the students of the rival schools.

During Long's student days, Dicken's "Pickwick Papers," "Oliver Twist" and "Nicholas Nickleby" were published. Thackeray, whose very name was unknown, was a contributor to Frazer's Magazine." Oliver Wendell Holmes, who later suggested the term, "anaesthesia," was trying for practice in Boston. Washington Irving was engaged in active literary work at his home, Sunnyside, in Tarrytown. Nathaniel Hawthorne was in Salem writing "Twice Told Tales." Motley was writing his first book, "Morton's Hope." The weird tales of the sombre genius, Edgar Allan Poe, were taking hold of the public imagination.

Longfellow was teaching modern languages in Harvard and writing "Hyperion." James Russell Lowell was a student at Harvard. Andrew Jackson was at the "Hermitage," in serene retirement after stressful and turbulent years.

The world, now recovered from the great conservative reaction which followed the French Revolution, was full of ferment, investigation, speculation and novel ideas.

Railroads were reaching out their tentacles on all sides, and the whistle of the locomotive had become the proclamation of civilization.

The steamboat "Great Western" had crossed the ocean from Bristol to New York in thirteen days and eight hours. Itinerant lecturers were showing to amused audiences the curious antics of persons who inhaled nitrous oxide gas, or, as it came to be called, laughing gas. Such exhibitions were called nitrous oxide frolics. Men were on the tip toe of expectation as to the supposed beneficial powers of hypnotism. It was learned with amazement that a hypnotized subject could feel no pain, and that Ward, in London, and Cloquet and Lysel, in France, had performed painless operations upon people sleeping the "magnetic sleep." Everybody felt that we were on the threshold of great events and that the first few, hesitating words of truth, had, as yet, but scarce been lisped by the baby lips of science.

Medical students must, of course, have heard of these things, discussed them with each other, asked questions of their professors and speculated as to the possibility of painless surgery. Every visit to the surgical clinic must have impressed on their minds the tortures inflicted by operations, and what a beneficent change it would be could a victim sleep under the knife. Neither Gibson, Wood, or Chapman had a word to say in favor of "animal magnetism," or Braidism, as it came to be called. Gibson's book says nothing at all about preventing pain in surgical operations. He certainly followed the usual custom—drugged the patient heavily with opium, and had him forcibly held or firmly strapped during the dread tragedy of the operation.

Nitrous oxide was a well-known drug and was lectured on by teachers of chemistry and therapeutics. Sir Humphrey Davy, in the year 1799, found out that, if inhaled, nitrous oxide would subdue pain, and suggested its use in surgical operations. In 1800 he published his experience and suggestion. Davy's recommendation was never acted on until Horace Wells used the gas as an anaesthetic in 1844. Hare taught that when nitrous oxide is inhaled, it produces "a transient, peculiar, various and generally vivacious ebriety."

Pareira, in his materia medica (1839), states that he had given nitrous oxide to about 100 persons, that it produces temporary and usually pleasing delirium, which subsides in three or four minutes—that the delirium takes different forms, causing some to dance and some to fight. In some few cases stupor is produced. He recommended it for spasmodic asthma.

Ether had been known for several centuries. Hare, in his chemistry, speaks of the internal administration of ether, but says nothing of the effects of inhalations. Wood does speak of ether inhalations. I find a reference to it in the Syllabus of his lectures. He says it may be inhaled, tells what inhalations are advised for, and explains how they are given. It was used in very small doses for spasmodic conditions. Dr. Wood states in his Therapeutics, written at a much later date than this, that "Ether has been long used by this method (inhalation). The late Dr. P. S. Physick was much in the habit of employing it in pulmonary affections, and invented a small, extemporaneous inhaler for the purpose." Dr. Physick died in 1838.*

Pareira discusses the stomach administration of ether, and says that large doses cause intoxication, and excessive doses, stupefaction. He also speaks of ether drinkers, and refers to a chemist suffering from cancer of the colon, who drank a pint of ether a day to relieve his pain. Pareira speaks of inhalation as follows: "When the vapour of ether, sufficiently diluted with atmospheric air, is inhaled, it causes irritation of the epiglottis, a sensation of fullness in the head and a succession of effects analogous to those caused by the protoxide of nitrogen, ————. If the air be too strongly impregnated with ether, stupefaction ensues. In one case this state continued, with occasional periods of intermission, for more than thirty hours: for many days the pulse was so much lowered that considerable fears were entertained for the safety of the patient. In another case, an apoplectic condition, which continued for nine hours, was produced." The case of lethargy for thirty hours, spoken of by Pareira, was originally referred to in an article published, in 1818, in the "English Quarterly Journal of Science and Arts," and supposed to have been written by Faraday.** Pareira was evidently fearful of the effects of ether by inhalation.*** He used it by dropping some of the drug on a lump of sugar and holding the sugar in the mouth, or by dropping ether in hot water and inhaling the vapour mixed with steam. It was recommended for chronic catarrh and dyspnoea, whooping cough, spasmodic asthma, and to relieve the effects produced by the accidental inhalation of chlorine.

*(To be Continued.)*

